# Targeting C–C Chemokine Receptor 5: Key to Opening the Neurorehabilitation Window After Ischemic Stroke

**DOI:** 10.3389/fncel.2022.876342

**Published:** 2022-04-28

**Authors:** Yi-Qi Feng, Zhen-Zhen Xu, Yan-Ting Wang, Yue Xiong, Wanli Xie, Yu-Yao He, Lu Chen, Guo-Yang Liu, Xia Li, Jie Liu, Qingping Wu

**Affiliations:** Department of Anesthesiology, Union Hospital, Tongji Medical College, Huazhong University of Science and Technology, Wuhan, China

**Keywords:** stroke, CCR5, chemokines, immune response, neuronal plasticity, neurorehabilitation

## Abstract

Stroke is the world’s second major cause of adult death and disability, resulting in the destruction of brain tissue and long-term neurological impairment; induction of neuronal plasticity can promote recovery after stroke. C–C chemokine receptor 5 (CCR5) can direct leukocyte migration and localization and is a co-receptor that can mediate human immunodeficiency virus (HIV) entry into cells. Its role in HIV infection and immune response has been extensively studied. Furthermore, CCR5 is widely expressed in the central nervous system (CNS), is engaged in various physiological activities such as brain development, neuronal differentiation, communication, survival, and learning and memory capabilities, and is also involved in the development of numerous neurological diseases. CCR5 is differentially upregulated in neurons after stroke, and the inhibition of CCR5 in specific regions of the brain promotes motor and cognitive recovery. The mechanism by which CCR5 acts as a therapeutic target to promote neurorehabilitation after stroke has rarely been systematically reported yet. Thus, this review aims to discuss the function of CCR5 in the CNS and the mechanism of its effect on post-stroke recovery by regulating neuroplasticity and the inflammatory response to provide an effective basis for clinical rehabilitation after stroke.

## Introduction

The limited recovery following acute brain damage leading to stroke is among the most common causes of adult physical disability worldwide (Benjamin et al., 2019), with ischemic stroke accounting for 71% of all strokes (Collaborators et al., 2018). Most ischemic strokes are thromboembolic in origin, and regions without adequate blood flow lead to energy depletion, metabolic disorders, and abnormal electrical activity, which become irreversibly injured and contribute to the clinical deficit over time (Campbell et al., 2019). Progress has been made in facilitating individuals’ recovery from ischemic stroke following advancements in pharmaceutical mechanic and thrombolysis (Paul and Candelario-Jalil, 2021). However, because of the limited window for reperfusion therapies and irreversible neuron death, approximately 50–60% of patients still suffer from motor impairments after successful endovascular clot removal (Schaechter, 2004; Leng and Xiong, 2019). Hence, apart from therapies that promote cerebral blood vessel reperfusion, medical treatments developed to enhance recovery after stroke have wide prospects for clinical application. The process of neuronal recovery in brain traumas, including stroke, includes upregulation of growth-promoting genes (Cramer and Procaccio, 2012), axonal sprouting (Li et al., 2010), and alterations in tonic gamma-aminobutyric acid and α-amino-3-hydroxy-5methyl-4-isoxazole propionic acid (AMPA) receptor signaling (Clarkson et al., 2010, 2011). Accumulating evidence indicates that enhancing plasticity processes in brain circuits plays a significant role in neurorehabilitation therapies after stroke (Joy and Carmichael, 2021).

C–C chemokine receptor 5 (CCR5) is a seven-membrane G protein-coupled receptor (GPCR) composed of 352 amino acids. CCR5 contains multiple ligands, including CCL3 (MIP-1α), CCL4 (MIP-1β), CCL5 (RANTES), CCL8 (MCP-2), CCL3L1 (LD78), and CCL11 (eotaxin; Chen et al., 2018; Jiao et al., 2019). Regulation of leukocyte migration is tightly linked to CCR5 expression and activation. In 1996, it was shown that CCR5 is key co-receptor that allows human immunodeficiency virus (HIV) to enter target cells (Alkhatib et al., 1996). Chemokine receptors, furthermore, play a role in a wide range of physiological and pathological processes in the central nervous system (CNS), as demonstrated by mounting evidence (Babcock et al., 2003; Rostène et al., 2007). In 2019, CCR5 was shown to be an effective therapeutic target for recovery from traumatic brain injury (TBI) and stroke, and became the first reported gene associated with enhanced recovery in human stroke (Joy et al., 2019). However, the mechanism by which CCR5 acts as a therapeutic target to promote neurorehabilitation after stroke has rarely been systematically reported. We have consequently reviewed the current knowledge of CCR5 distribution and function, as well as its mechanism and research development, as a target for ischemic stroke rehabilitation.

## C–C Chemokine Receptor 5 in the Body: Distribution and Functions

### C–C Chemokine Receptor 5 Expression and Localization

Chemokine receptors are critical for immune cell recruitment and development and play important roles in a wide range of inflammatory responses, both protective and destructive. CCR5 expression has been demonstrated in various immune cells, including dendritic cells, NK cells, macrophages, T-lymphocytes, and B-lymphocytes (Griffith et al., 2014; Hughes and Nibbs, 2018). In addition, microglia, astrocytes, and neurons in the CNS also express CCR5 (Klein et al., 1999; Mennicken et al., 2002; Westmoreland et al., 2002; Flynn et al., 2003). The presence of CCR5 in vascular smooth muscle cells and capillary endothelial cells has also been reported, but its function is still poorly defined (Rottman et al., 1997; Murphy et al., 2000; Jones et al., 2011). Table 1 shows the expression of CCR5 gene in cells.

**TABLE 1:** Expression of CCR5 genes in cells.

System	Cell type	Species	Author and year
The cardio-vascular system	Vascular endothelial cells (coronary endothelia, brain endothelia)	Human	Berger et al., 1999
	Vascular smooth muscle cells (aorta, coronary artery and saphenous vein)	Human, Macaques	Rottman et al., 1997
The central nervous system	Neurons (CA1–4 pyramidal hippocampal, dentate gyrus, cortical white matter, brain stem neurons)	Human, Macaques	Westmoreland et al., 2002
	Astrocytes	Human	Flynn et al., 2003
	Microglia		
The immune system	**Innate lymphocytes**	Human	Hughes and Nibbs, 2018
	Innate lymphoid		
	Natural killer cells		
	**Myeloid cells**		
	Mactophages (spleen, lung, body cavity, intestine, liver) classical/non-classical monocytes (bone marrow, blood)		
	**Dendritic cells (DCs)**		
	Skin DCs/Langerhans cells		
	DCs (thymus, spleen, lymph node, skin, lung)		
	DC precursors		
	**B/T-lymphocytes**		
	Natural killer T cells		
	Marginal Zone B cells		
	B cell precursors (bone marrow)		
	Memory CD4^+^/CD8^+^ T cells		
	Activated CD8^+^ T cells		
	B1 cells		
	γσ T cells (thymus, periphery)		

### Signaling Pathways in C–C Chemokine Receptor 5 Functions

**FIGURE 1:**
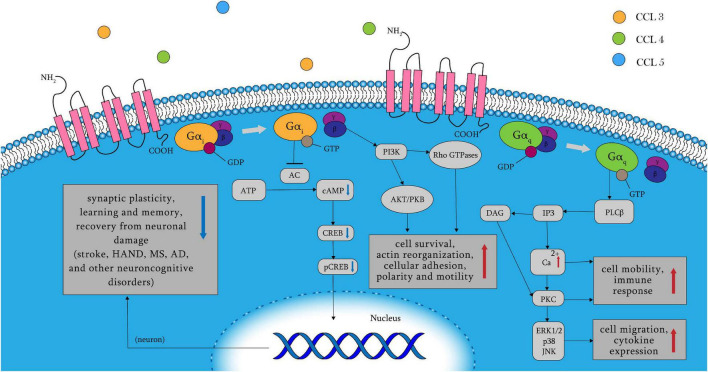
CCR5 receptor signaling pathways. When the amino terminus of CCR5 binds to its ligand, its following signaling pathway is mainly controlled by Gα_*q*_, Gα_*i*_, and βγ subunits. The Gα_*i*_ pathway results in the release of intracellular Ca^2+^ and decreased learning and memory function, as well as worsened recovery from neuronal damage by reduced cAMP and pCREB. Activation of Gα_*q*_ results in cell migration and cytokine expression in immune response. And the βγ subunits leads to cell survival, adhesion and motility through multiple following signaling pathways such as AKT/PKB and Rho GTPase. AC, adenylyl cyclase; AD, Alzheimer’s disease; AKT/PKB, protein kinase B; cAMP, cyclic adenosine monophosphate; CCR5, C–C chemokine receptor 5; CREB, cAMP-response-element binding protein; DAG, diacylglycerol; HAND, HIV-related neurocognitive disorders; IP3, inositol 1,4,5-trisphosphate; JNK, C-Jun N-terminal kinases; MS, multiple sclerosis; pCREB, phosphorylated cAMP-response-element binding protein; PKB/Akt, protein kinase B; PKC, protein kinase C; PLC, phospholipase C.

C–C chemokine receptor 5 belongs to the GPCR family and, contains multiple ligands, including CCL3, CCL4, and CCL5. Other inflammatory chemokines that act as CCR5 agonists include CCL8, CCL3L1, and CCL11 (Zlotnik and Yoshie, 2012; Bredesen, 2014; Brelot and Chakrabarti, 2018). Binding of ligands to CCR5 leads to the dissociation of the G protein heterotrimer into α and βγ subunits, and the α subunits include two types: Gα_*q*_ and Gα_*i*_ (Figure 1). When Gα_*q*_ is activated, PLC, which splits PIP2 into IP3 and DAG, is activated. Calcium levels are raised by IP3, which triggers PKC with DAG. The release of intracellular Ca^2+^ activates the mitogen-activated protein (MAP) kinases ERK1/2 (extracellular signal-regulated kinase), p38, and JNK, which paly essential roles in cell migration and immune response, as well as proline-rich tyrosine kinase 2, which is important for cell motility (Dairaghi et al., 1998; Ganju et al., 1998; Del Corno et al., 2001; Kraft et al., 2001; Missé et al., 2001; Wong et al., 2001; Brelot and Chakrabarti, 2018). In addition, the Gα_*i*_ pathway inhibits adenylyl cyclase, resulting in decreased cAMP and pCREB levels. As a result, plasticity-related protein transcription and synaptic plasticity are reduced, leading to poorer learning and memory function, as well as a worsened recovery from neuronal damage. Neuronal plasticity and memory problems have been linked to stroke, Huntington’s disease, Alzheimer’s disease, and other neurocognitive disorders (Zhou et al., 2009; Sano et al., 2014; Lorenzen et al., 2018). Rho GTPase and protein kinase B (PKB/Akt) are stimulated when βγ subunits activate PI-3K. The former is associated with cell survival, whereas the latter regulates cell adhesion and motility (Burgering and Coffer, 1995; Neptune and Bourne, 1997; Downward, 2004; Oppermann, 2004). CCR5 activation also results in phosphorylation of Janus kinases (JAK2) and subsequent activation of the JAK/STAT pathway, which is unaffected by Gα_*q*_ or Gα_*i*_ (Mueller and Strange, 2004). CCR5 is involved in a variety of cellular biological changes through complex signaling processes, including cell migration, adhesion, survival, and neuronal plasticity.

### Physiological Function of C–C Chemokine Receptor 5

#### Functions of C–C Chemokine Receptor 5 in Immune System

##### C–C Chemokine Receptor 5 Induces Immune Cell Migration

Leukocyte motility plays a critical role in inflammatory responses, as it is essential to rapidly recruit innate immune cells to kill pathogens, prevent the spread of microorganisms, trigger inflammation, and repair damage. The classical function of chemokines is to regulate inflammation and drive immune cells through the blood and lymphatic vessels, directing their migration and localization by forming soluble concentration gradients in an autocrine or paracrine manner (Hughes and Nibbs, 2018; Lau et al., 2020). In some contexts, chemokines are also selectively recruited to the cell surface by glycosaminoglycans polysaccharides present within extracellular matrices and at the cell surface which can form fixed concentration gradients and fine-tune the immune response (Paul and Candelario-Jalil, 2021). In NK cells and mast cell precursors, CCR5 binds to locally generated chemokines and inflammatory mediators and causes these innate immune cells to cross the endothelium into peripheral tissues during acute inflammation. Chemokines can regulate immunological responses by guiding regulatory T cell (Treg) migration, and CCR5 may influence the antibody response in the lymph nodes by enhancing the contact of Tregs with B cells and antigen presenting cells (APCs; Maurer and von Stebut, 2004; Khan et al., 2006; Maghazachi, 2010; Griffith et al., 2014). In addition, CCL3/CCR5 was shown to be very effective in augmenting the adhesion of the monocytes to intercellular adhesion molecule-1 during monocyte–endothelial cell interactions, and CCL4/CCR5 can enhance the adhesion of T lymphocytes to vascular cell adhesion molecule 1, which drives immune cell migration from the blood to local tissues across the endothelium (Menten et al., 2002).

##### C–C Chemokine Receptor 5 Affects Immune Cell Differentiation

Chemokines can induce T cells to differentiate into different subsets and mediate different types of immune responses. Interferon production and macrophage activation are associated with the T helper (Th)1 response; whereas antibody response, B cell assistance, and IL-4 and IL-5 production are associated Th2 response. CCL3, CCL4, and CCL5, which are ligands of CCR5, are chemotactic on Th1 cells, but not on Th2 cells; Th2 cells mainly express CCR2 and CCR4 (Rossi and Zlotnik, 2000; Wong and Fish, 2003). There was a skewed Th2 cytokine profile in mice lacking CCR5, indicating that CCL3/CCR5 and CCL4/CCR5 can influence the immune response by regulating the differentiation of Th to Th1 (Andres et al., 2000; Luther and Cyster, 2001).

##### C–C Chemokine Receptor 5 Promotes Immune Cell Activation

Binding of CCR5 to ligands causes the CD8^+^ subset of DC to produce IL-12, which is regarded as a critical step in initiating cell-mediated immunity against intracellular infections (Aliberti et al., 2000). CCR5 is activated when T cells come into contact with APCs and is induced by IL-2 into positive feedback expression on T cells. Simultaneously, CCR5 is recruited to the immunological synapse to reduce T cell responsiveness to other chemotactic substances, through which the stability of T cell-APC interactions is increased and T cell activation is enhanced (Molon et al., 2005). When CD4^+^ T cells interact with DCs, both CD4^+^ T cells and the DC generate CCL3 and CCL4. At the same time, CCR5 is upregulated on naive CD8^+^ T cells entering the lymph node and binds to these chemokines, promoting their migration to CD4^+^ T cell/DC clusters, which finally leads to improved interactions between naive CD8^+^ T cells and DCs licensed by CD4^+^ T helper cells and increased quality and quantity of the CD8^+^ T cell memory response (Del Corno et al., 2001; Griffith et al., 2014). Bystander T cells in the immune system can rapidly respond and secrete cytokines even without antigenic stimulation. CCL5/CCR5 may promote cytokine production and proliferation of bystander T cells, which is important for autoimmunity (Wong and Fish, 2003).

##### C–C Chemokine Receptor 5 Regulates Immune Cell Survival and Apoptosis

The goal of the host’s reaction to intracellular infections is to eliminate the infected cells as quickly as possible. In addition, it is crucial to remove infected, apoptotic cells from the tissue. Pathogens, activated macrophages, and residual apoptotic cells through proinflammatory proteinases and cytokines cause further tissue damage if the clearance process is disrupted. CCL5-CCR5 interaction can activate MEK-ERK and PI3K-AKT anti-apoptotic signals, which mediate cell growth and survival (Tyner et al., 2005). CCR5 may stimulate T-cell proliferation by triggering STATs (signal transducers and activators of transcription) activation, as chemokine receptors can regulate many transcription factors (López-Cotarelo et al., 2017). By binding to and activating CCR5, CCL5 generated by melanoma tumor cells may trigger the death of tumor-infiltrating T lymphocytes. This process is dependent on the release of cytochrome c into the cytoplasm, rather than on the Fas/Fas ligand. Continued activation of CCR5 by CCL5 following T-cell activation has been shown to cause T-cell death in other studies, indicating that it is a chemokine-dependent late regulation mechanism at an inflammatory site (Wong and Fish, 2003).

#### Functions of C–C Chemokine Receptor 5 in Central Nervous System

Chemokines, including astrocytes, microglia, and neurons, are expressed in the human CNS from the embryonic stage to adult stage (Westmoreland et al., 2002). CCR5 is mainly distributed in microglia, with lower expression levels in astrocytes and neurons, although all are upregulated in disease states (Xia et al., 1998; Cartier et al., 2005). Numerous experiments have demonstrated that chemokines in the brain function beyond directing immune cell migration, including brain development, neuronal differentiation, neuronal communication, neuron survival, and learning and memory capabilities, directly by affecting neurons, or indirectly through glial cells (Song et al., 2002; Sorce et al., 2011; Wang et al., 2016).

##### C–C Chemokine Receptor 5 Is Involved in Brain Development and Neuronal Differentiation

Neural progenitor cells express CCR5 and are significantly induced to migrate by CCL5 from activated microglia and astrocytes during human brain development; this migration can be suppressed by antibodies against CCR5 (Park et al., 2009). Mutant CCR5 mice showed fewer and later differentiation of neuronal cells with instant motor deficits, and lacked nociceptive responses, this supports the idea that CCR5 participates in the development of the CNS by inducing neural progenitor cells to migrate to their target destination and promoting neuronal differentiation (Aarum et al., 2003; Tran et al., 2007; Park et al., 2009). CCR5 KO mice have fewer nigral dopaminergic neurons than normal mice, suggesting that CCR5 may play an important role in promoting maturation or development of the nigral dopaminergic system (Park et al., 2009; Choi et al., 2013). Furthermore, the recruitment function of CCR5 promotes monocyte migration along the hippocampal sulcus during brain development and facilitates microglial colonization of the nervous system. Under normal conditions, attracting microglia by chemokines produced by neural progenitor cells may play an essential role in normal brain function, including nutritional support, regulation of neuronal development, and removal of toxic debris (Polazzi and Contestabile, 2002; Ji et al., 2004; Simard et al., 2006; Hahn et al., 2010). Simultaneously, CCR5 ligands from progenitors, microglia, and astrocytes can attract CCR5-expressing dendritic cells, lymphocytes, and monocytes across the immature blood-brain barrier (BBB) into the CNS to inspect the newly generated cells (Cowell et al., 2006; Whitney et al., 2009). CCR5 activation can regulate many transcription factors including cAMP-response-element binding protein (CREB), which participates in a variety of cellular activities and plays an essential role in the CNS, such as neuronal development, neuroprotection, and disease processes (Finkbeiner, 2000; Lonze and Ginty, 2002; Kuipers et al., 2008). These findings may partially explain the function of CCR5 in brain development and neuronal differentiation; however, the implications of these findings remain unclear.

##### C–C Chemokine Receptor 5 Affects Signaling Between Neuronal Cells

By sensing chemokines in the blood generated by immune cells, area postrema (AP)/nucleus tractus solitarius neurons in the CNS may deliver signals from the active immune system to the CNS. Through voltage-dependent Ca^2+^ channels, chemokine receptors can reduce Ca^2+^ influx so that Ca^2+^-dependent K^+^ currents might be inhibited, thereby regulating neuronal excitability and neurotransmitter release (Oh et al., 2002). However, the activation of presynaptic chemokine receptors on hippocampal neurons can regulate the release of glutamate at these synapses and reduce the frequency by voltage-dependent Ca^2+^ channels; this may produce presynaptic inhibition (Meucci et al., 1998). The current study shows that because of PLC/IP3-induced Ca^2+^ mobilization following G protein activation, CCL5/CCR5 plays a dual role in glutamate transmission: the chemokine inhibits the depolarization-evoked glutamate release, but potentiates the basal release of glutamate (Musante et al., 2008).

##### C–C Chemokine Receptor 5 Regulates Neuronal Survival and Apoptosis

C–C chemokine receptor 5 regulates a variety of transcription factors involved in cell survival such as STAT and CREB. Furthermore, because CCR5 mediates crosstalk between glia and neurons, it is critical for neuronal survival in both normal and pathological situations (Choi et al., 2013). Various stimuli may induce the expression of CCR5, such as proinflammatory cytokines TNF-α and INF-γ, lipopolysaccharide (LPS), and hypoxic-ischemic brain injury; these can lead to neuronal death through the release of excitatory amino acids and reactive oxygen species (Hagberg and Mallard, 2005; Kaul et al., 2005; Cowell et al., 2006). During brain aging and neurodegeneration, the function of plasma membrane Ca^2+^-ATPase gradually declines, and a large amount of cytosolic Ca^2+^ released via the CCL5/CCR5 and PLC/IP3 pathways cannot be effectively removed. Inflammatory CCL5 activity and long-lasting Ca^2+^ dyshomeostasis can lead to neuronal apoptosis (Radzik et al., 2019). In addition, chemokines regulate the production of matrix metalloproteinases (MMPs), suggesting that they may contribute to MMP activity-mediated neuronal cell survival and death (Rostène et al., 2007).

##### C–C Chemokine Receptor 5 Inhibits Learning and Memory Processes

As a strong inhibitor of hippocampal and cortical plasticity, CCR5 affects the MAPK/CREB signaling pathway to influence learning and memory. During learning, MAPK and CREB levels are enhanced after CCR5 antagonist use, region-specific viral knockdown, or CCR5 knockout, whereas transgenic mice that overexpress CCR5 in excitatory neurons display learning and memory deficits (Zhou et al., 2016; Merino et al., 2020). The transcription factor CREB in neurons promotes long-term potentiation and enhances synaptic plasticity. Simultaneously, neurons with higher CREB levels are more excitable; consequently, they are more likely to be recruited to participate in the process of learning and memory (Zhou et al., 2009). N-methyl D-aspartate receptor 1 (NMDAR-1) plays an important role in social recognition behavior, as demonstrated by the impaired social recognition of mice with inactive NMDAR-1 receptors. The expression of NMDAR-1 is higher in the brains of CCR5^–/–^ mice than in those of WT mice; additionally CCR5^–/–^ mice show a significant improvement in social identification (Kalkonde et al., 2011). Dendritic spine turnover and spine clustering associated with learning and memory depend on NMDAR. In mammals, higher pre-learning spine turnover rates are closely linked to increased levels of learning and memory, which occur during important developmental periods as well as maturity. After CCR5 knockout, increased dendritic spine turnover rates may allow neurons to explore this space more frequently, enhancing connections with appropriate presynaptic neurons and consolidating new synapses through clustering during learning (Frank et al., 2018). CCR5 activation can lead to impaired AMPA-dependent synaptic transmission and significantly reduced excitatory postsynaptic potential, thereby impairing long-term memory and cognitive deficits (Marciniak et al., 2015).

Additionally, astrocytes involved in learning and memory processes can secrete chemokines to mediate synaptic transmission and plasticity after binding to CCR5 via various mechanisms; these including prevention of extrasynaptic neurotransmitter diffusion and removal to modulate synaptic release (De Pitta et al., 2016; Necula et al., 2021).

##### C–C Chemokine Receptor 5 Mediates Neuroinflammation

C–C chemokine receptor 5 participates in the inflammatory response in the CNS not only by mediating immune cell migration but also by affecting the permeability of the BBB and activating microglia (Necula et al., 2021). DCs can acquire essential maturation signals from invading T cells when inflammation occurs around CNS blood arteries. They then operate locally to boost immunological responses or trigger additional waves of autoreactive T cells after traveling to draining lymph nodes (Ambrosini et al., 2005). In the early stages of inflammation, the chemokine CCL5 released from endothelial cells induces resident brain microglia expressing CCR5 to migrate to cerebral vessels, causing microglial cells to infiltrate through the neurovascular unit and express CLDN5. Thus brain microglia initially contact endothelial cells and develop tight junctions to maintain the integrity of BBB. However, prolonged inflammation may cause brain microglia to evolve into a phagocytic phenotype that includes morphological alterations, astrocytic fragment engulfment, and leakage across the BBB. Therefore, CCR5-positive microglia play a dual role in inflammation-induced BBB permeability (Haruwaka et al., 2019). The migration of CCL5-driven peripheral blood mononuclear cells across the BBB is dependent on ligand interactions with CCR1 and CCR5: CCR1 is involved in the arrest, while CCR5 is involved in spreading. These interactions can directly affect the development of certain neuroinflammatory diseases (Ubogu et al., 2006). At the same time, CCR5 activation and Ca^2+^ increase can affect gene expression and microglia activation, which can promote microglial cell activation and proliferation. Therapeutic targeting of CCR5 may decrease BBB leakage, increase neurogenesis stimulated by the excitotoxin kainic acid (KA), and promote migration of bone marrow-derived cells to the brain to become neurons, thereby promoting the repair of nervous system damage (Louboutin and Strayer, 2013).

##### C–C Chemokine Receptor 5 Mediates the Interaction Between Neurons and Glial Cells

UsingCCR5 and its ligands, bidirectional interactions between neurons and between neurons and glial cells play a critical role in maintaining normal neuronal activity (Choi et al., 2013). The CCR5/CREB pathway affects plasticity in neurons in a unique way, whereas CCR5 activation in glial cells is intimately linked to elevated Ca^2+^ (Louboutin and Strayer, 2013). Microglia can communicate with immune cells and neurons through a variety of signaling pathways. Microglial cells undergo a complex, multistage activation process, when evidence of brain lesions or nervous system dysfunction is detected, which allows them to migrate to the injury site, phagocytose cells, proliferate, and lead to protective or neurotoxic effects by releasing active substances (Gebicke-Haerter, 2001; Streit, 2002). CCR5 participates in the migration and activation of microglia through Ca^2+^ signaling, which may damage vascular epithelial cells and neurons (Shideman et al., 2006). Furthermore, a GPCR screen showed the role of CCR5 in microglial neurotoxicity suppression; it is activated by RANTES, as a signal mediator between microglia and neurons, and decreases the expression of toxic iNOS and inflammatory cytokines (Gamo et al., 2008). Beyond being a component of the immune response, microglia also can rebuild dendritic spines and synaptic adhesion and transmission after CCR5-mediated migration to ligand-directed chemotactic gradients, which leads to network-level effects (Ekdahl, 2012; Louboutin and Strayer, 2013; Posfai et al., 2019). Astrocytes also respond to a variety of stimuli via CCR5 mediated calcium signals, supporting and modulating particular neuronal networks in different ways after activation by ligands (Ben Haim and Rowitch, 2017). Additionally, astrocytes, which are involved in learning and memory processes, can secrete chemokines to mediate synaptic transmission and plasticity after binding to CCR5 via various mechanisms; these include the prevention of extrasynaptic neurotransmitter diffusion and removal to modulate synaptic release (De Pitta et al., 2016; Adamsky et al., 2018; Allen and Lyons, 2018).

## C–C Chemokine Receptor 5 and Stroke

### Effects of C–C Chemokine Receptor 5 Inhibition on Neurological Rehabilitation After Stroke

After a stroke, multiple biochemical and molecular mechanisms can cause brain damage. The activation and interaction of different signaling pathways following ischemia have different effects on the final extent of the infarct. Inducible upregulation of CCR5 and its ligands after stroke is particularly pronounced in neurons. There are many relevant studies on the effect of CCR5 inhibition after stroke, but owing to the redundant manner in which chemokine families act, complex cellular and molecular changes after stroke, and differences in experimental methods and genetic backgrounds, the results of these studies vary to some extent. In conclusion, mice knocked out for the CCR5 gene showed increased neuronal apoptosis and increased infarct size in histological analysis within 1 week after cerebral ischemia. These results may be explained by the increased expression of CCR2 caused by the loss of CCR5, which activates inflammatory response and increases neuronal degeneration and apoptosis (Zhou et al., 2009; Sorce et al., 2010). CCR5 activation is also required for adoptively the adoptive transfer of Tregs to the ischemia-damaged endothelium. Tregs can prevent proteolytic damage to the BBB by inhibiting the production of matrix metallopeptidase 9. Therefore, CCR5 deficiency may contribute to BBB damage and increased inflammation after stroke (Li et al., 2017). However, it is clear that CCR5 has important effects on neurological recovery in the subacute phase after stroke, because mice injected with shCCR5 adeno-associated virus into the pre-motor cortex showed a significant and sustained improvement in motor control and cognitive function 1 week after stroke (Joy et al., 2019). This improvement does not occur through neuronal protection, and it mechanism is elaborated below. Furthermore, 2 months after cortical ischemia, brain-derived CCR5 deficiency causes an increase in infarction size, dendritic loss in the peri-infarct cortex, and less long-term inflammatory cell accumulation. This discrepancy shows that the role of brain-derived CCR5 in preserving and regulating neurostructural connections after stroke may be cell dependent (Ping et al., 2021).

A 32-bp deletion in CCR5 causes the receptor to be non-functional. Furthermore, the homozygous CCR5 delta32 deletion confers inherent resistance to HIV infection, which is found in approxiamately 1% of Caucasians (Hutter et al., 2009). The CCR5-Δ32 mutation is the first report of a human genetic variable linked to enhanced stroke recovery. Patients with loss-of-function CCR5 were found to have enhanced stroke recovery on multiple measures of motor, cognitive, and sensory function in a large patient cohort, which included verbal functioning, memory, and attention (Ben Assayag et al., 2012). Post-stroke depression is a common neuropsychiatric comorbidity, it may negatively affect outcomes by increasing the rates of disability and mortality (Wu et al., 2018). A clinical study showed that, compared with non-carriers, depressive symptoms tend to improve over time after stroke in CCR5-Δ32 carriers, which provides further evidence that inhibition of CCR5 function is a protective factor for neurorehabilitation after stroke (Tene et al., 2021).

### Changes of Cell Biology After C–C Chemokine Receptor 5 Inhibition

#### Inflammatory Response in Central Nervous System Is Reduced

In addition to adhesion molecules and inflammatory cytokines, chemokines and their receptors play critical roles in the accumulation of leukocytes around the infarct tissues (Feuerstein et al., 1998). Local and peripheral immune cells, including astrocytes, microglia, neutrophils, macrophages, and monocytes, are recruited after a stroke (Iadecola and Anrather, 2011; Chen et al., 2020). Inhibition of CCR5 expression in premotor cortical neurons after stroke can modulate astrogliosis, reduce astrocyte reactivity, and dampen macrophage recruitment; this inhibition as an intervention can help create a conducive environment for neural repair (Adelson et al., 2012; Barreto et al., 2012; Liraz-Zaltsman et al., 2021). CCR5 inhibition of premotor cortical neurons has no significant effect on microglia responsiveness, but 2 months after ischemia, mice with complete CCR5 deletion in the brain have fewer long-term inflammatory cells, such as Iba1^+^ cell infiltration around the peri-infarct tissues (Sorce et al., 2010; Ping et al., 2021).

#### Neuronal Excitability Is Increased

Neuronal excitability and plasticity are similar mechanisms between normal memory formation and recovery after stroke, which can strengthen the connections underlying memory formation and restore lost motor function after stroke. CCR5 inhibition increases neuronal CREB and pCREB expression after stroke, thereby enhancing cellular excitability (Kandel, 2012; Joy et al., 2019). After stroke, there were four separate time epochs: hyperacute, acute, subacute, and chronic (Bernhardt et al., 2017). In the hyperacute phase of stroke, extensive cell death occurs, followed by an acute phase of delayed neuronal apoptosis 1 week later, during which increased neuronal excitability can exacerbate brain damage due to enhanced excitotoxic signaling pathways (Joy and Carmichael, 2021). The subacute period of stroke lasts approximately a month in rodents and up to 3 months in humans, and increased neuronal excitability during this phase can promote motor recovery (Cheng et al., 2014), which partly explains the differential effect on brain injury after CCR5 inhibition. Furthermore, activation of CCR5 affects glutamate release and may affect neuronal signaling through AMPA receptors. Although there are no clear related studies, inhibition of CCR5 may alter neuronal excitability by modulating excitatory neurotransmitters and signaling (Musante et al., 2008; Marciniak et al., 2015). Similar to CCR5, the AMPA receptor is important in the pathophysiology of stroke, but is functionally contradictory. Binding of glutamate to AMPA receptors results in cation influx, depolarization, and the expression of downstream genes, such as brain-derived neurotrophic factor (BDNF). Early potentiation of AMPAR signaling exacerbates stroke damage, while delayed enhancement of the same system may enhance functional recovery (Jourdi et al., 2009; Clarkson et al., 2011). Because both initial cell death and delayed restorations of function are caused by excitatory pathways in neurons, CCR5-targeted treatment must be administered at specified time intervals after the beginning of the stroke.

#### Neuronal Plasticity Is Increased

In the subacute phase of stroke, the brain is in a plastic state called the sensitive period in stroke recovery, similar to the critical period of enhanced plasticity during development (Carmichael, 2016; Zeiler et al., 2016). Increased plasticity during this period leads to axonal sprouting, dendritic spine morphogenesis, and the remapping of movement representations, which are extremely important for the recovery of adult brain injury (Li et al., 2010; Cirillo et al., 2020). During the sensitive period of stroke recovery and learning and memory, common mechanisms include neuronal distribution, competitiveness controlled by the excitability state, morphogenesis of dendritic spine in engram generation, and compensation for disrupted engrams. After CCR5 inhibition in cortical neurons, enhanced CREB signaling increases neuronal excitability. Furthermore, neurons with higher excitability are more likely to synergize with other neurons after the arrival of a stimulus, forming an engram that will be stored (Yiu et al., 2014). Specifically, because of increased CREB function, neurons with CCR5 knockdown are specifically incorporated into the same motor circuit. CCR5 knockdown induces upregulation of CREB and downstream proteins, such as dual-leucine zipper kinase proteins, in the premotor cortex, which may help preserve the dendritic spine in the early stages of stroke, induce axonal sprouting in the contralateral cortex, improve remapping of damaged sensory and injured motor circuits, and stimulate the creation of new links in these circuits (Joy et al., 2019). Furthermore, CREB induction can also adaptive or compensatory if nearby areas of the brain are injured because of the compensation of memory engrams for brain regions that are dysfunctional or inactivated (Caracciolo et al., 2018). In addition, CCR5 may indirectly affect the plasticity of the CNS after stroke by affecting the reactivity of astrocytes. Activated astrocytes can form glial scars after ischemic injury, which sequesters the injury site and protects cells against the release of harmful substances during the acute phase (Liu and Chopp, 2016). However, persistent glial scars hinder axonal regeneration and inhibit neural plasticity (Pekny et al., 2016). Thus, decreased astrocyte reactivity after CCR5 inhibition may be beneficial to functional outcomes.

### Clinical Significance of Targeting C–C Chemokine Receptor 5 After Ischemic Stroke

Additionally, treatment with Maraviroc (Pfizer, New York, United States), a CCR5 antagonist approved by the FDA, improves motor recovery in rodent models of stroke and TBI by enhancing tissue preservation in the brain, attenuating inflammatory responses, and upregulating the levels of cognition-related signaling molecules to promote neural plasticity (Villanueva, 2019; Friedman-Levi et al., 2021). However, using antagonists to inhibit the function of CCR5 requires improved consideration of its potential negative effects. In other studies, CCR5-deficient mice showed increased damage after stroke, which is mainly related to differences in animal models and time of intervention (Sorce et al., 2010). Knockout of the CCR5 gene, compared with using Maraviroc and other antagonists, or small interfering RNA, may cause dramatic effects on cell signaling pathways and lead to a worse prognosis, while reducing CCR5 expression in specific cell types may be more beneficial for neurorehabilitation (Joy et al., 2019; Ping et al., 2021). Furthermore, the enhancement of neuronal excitability after inhibition of CCR5 increases neuronal death in the acute phase; therefore, CCR5-targeted treatment at a specified time in the late subacute phase may be more beneficial for ischemic stroke (Clarkson et al., 2011; Joy and Carmichael, 2021). Although it plays multiple roles represented by immunology in the CNS, inhibition of CCR5 during pathological injury does not reduce immune microglial migration, which notes the loss of CCR5 may be compensated by increased expression of CCR3 and CCR2 (Bauss et al., 2021). Therefore, due to the complexity and interactions of the chemokine receptor family, the benefits of CCR5-targeted therapy after stroke require further investigation and confirmation. Maraviroc is currently being tested in clinical trials for stroke recovery (NCT03172026; Medicine, 2019).

In addition to promoting neurorehabilitation, recent studies have suggested other clinical implications of CCR5 in the context of stroke, including identification of stroke type and prognosis. Transient ischemic attack and ischemic stroke together constitute ischemic cerebrovascular disease, which has four subtypes: cardioembolism, large artery atherosclerosis, cryptogenic disease, and small artery occlusion. A study showed that in cardioembolism but not in other subtypes, the Δ32 allele frequency was lower, which suggests that CCR5 Δ32 plays a protective role in the cardioembolism, and Δ32 polymorphism helps identify stroke type (Kostulas et al., 2009). CCR5^+^ Tregs combined with Tregs may function as biomarkers for predicting the prognosis of ischemic stroke. CCR5 chemotactic Tregs can reduce inflammation after stroke and protect the BBB. Higher expression of Tregs often appears in severe stroke patients and large infarction groups, and high expression of CCR5^+^ Tregs may indicate mild stroke and smaller infarct area (Zhang et al., 2020).

## C–C Chemokine Receptor 5 and Other Diseases

C–C chemokine receptor 5 is involved in the pathophysiological process of a wide range of human diseases through its complex signaling pathways, ranging from infectious diseases, tumors to various neurological diseases, and its mechanism in different diseases may also provide clues for targeted rehabilitation therapy after stroke. Table 2 summaries CCR5’s different roles in CNS during normal and disordered conditions.

**TABLE 2:** Various roles of CCR5 in the CNS during healthy and diseased states.

Condition	Cell type	Role of CCR5 in CNS	References
Normal	Neural progenitor cells	Induces neural progenitor cells to migrate and promotes neuronal differentiation	Ji et al., 2004; Park et al., 2009
	Neuron	Promotes neuronal growth and differentiation during embryonic development	Bolin et al., 1998; Park et al., 2009
		Modulates neuronal excitability and neurotransmitter release	Oh et al., 2002; Musante et al., 2008
		Regulates neuronal survival or apoptosis	Choi et al., 2013; Radzik et al., 2019
		Inhibits hippocampus and cortical plasticity, impairs learning and memory processes	Zhou et al., 2016; Necula et al., 2021
	Microglia	Mediates microglia migration and colonization, which may provide nutritional support, regulation of neuronal development, and removal of toxic debris	Polazzi and Contestabile, 2002; Cowell et al., 2006
	Astrocytes	Regulates the proliferation, survival and differentiation of astrocyte progenitors during embryonic development	Bakhiet et al., 2001; Cartier et al., 2005; Necula et al., 2021
Ischemic stroke	Neuron	Inhibits neuronal plasticity and recovery	Joy et al., 2019; Joy and Carmichael, 2021
	Astrocytes	Promotes astrocytes proliferation and activation	Liraz-Zaltsman et al., 2021
	Monocytes	Recruites monocytes and modulates the immune response	Cartier et al., 2005
	Treg cells	Mediates the docking of transferred Tregs to relieve neutrophil accumulation and protect BBB	Sorce et al., 2010; Li et al., 2017
	Neutrophils	Induces neutrophils migration toward the injured area and leads to deteriorated brain injury	Chen et al., 2020
Intracerebral hemorrhage	Neuron	Mediates neurological deficits and neuronal pyroptosis via CCR5/PKA/CREB pathway	Yan et al., 2021
Neuroinflammation	Microglia	Induces microglia proliferation and activation	Louboutin and Strayer, 2013
	Monocytes	Mediates monocyte migration and affects the leakage of BBB	Ubogu et al., 2006; Louboutin et al., 2011
**Infectious disease**			
West Nile virus infection	Monocytes, Macrophages, NK cells, and T lymphocytes	Regulates trafficking of leukocytes to CNS to contain and clear the virus	Glass et al., 2005
Mouse Hepatitis Virus intracranial infection	Macrophages	Mediates macrophage trafficking into CNS and leads to demyelination	Glass et al., 2001
HIV-Associated Neurocognitive Disease (HAND)	CD4^+^ T lymphocytes	Mediates HIV-1 entry into CD4^+^ cells as a fusion cofactor	Kwong et al., 1998
	Microglia	Promotes microglial activation and neuronal damage, thereby improved cognition	Maung et al., 2014; D’Antoni et al., 2018
	Monocytes	Recruits HIV-infected monocytes to CNS and leads to intracranial infection and inflammation.	Hahn et al., 2010
Cerebral malaria	CD8^+^ T lymphocytes	Regulates trafficking of CD8^+^ T lymphocytes to destroy brain endothelial cells and BBB	Belnoue et al., 2003
**Cancer**			
Primary central nervous system lymphomas	Malignant B lymphocytes	Induces B lymphocytes homing to the brain and spreading within CNS	Brunn et al., 2007
Glioblastoma	Glioma-associated microglia/macrophages (GAMs)	Induces glioma invasive process	Yu-Ju Wu et al., 2020
		Regulates M1/M2 microglia phenotype	Laudati et al., 2017
	Treg cells, Monocyte	Recruits immunosuppressive cells to induce immune tolerance	Jiao et al., 2019
	Tumor cell	Promotes tumor cell proliferation and migration	Aldinucci and Colombatti, 2014
Pathological pain	Neuron	Activates neuron ERK to create and maintain pathological pain	Piotrowska et al., 2016; Hang et al., 2017; Lu et al., 2017
		Reduces the antinociceptive action of opioid receptor agonists	Szabo et al., 2002; Chen et al., 2007
Multiple sclerosis (MS)	T lymphocytes	Regulates trafficking of inflammatory T cells into CNS to induce self-destructive inflammatory process	Zang et al., 2000; Menten et al., 2002; Schlager et al., 2016
**Neurodegenerative disease**			
Alzheimer’s disease (AD)	Microglia, Astrocytes	Recruits and activates astrocytes and microglia to affect amyloid deposition and memory function with CCR2	Cartier et al., 2005; Lee et al., 2009; Goldeck et al., 2013
Parkinson’s disease (PD)	neuron	Promotes maturation of nigral dopaminergic neurons	Choi et al., 2013

### C–C Chemokine Receptor 5 and Infectious Diseases

C–C chemokine receptor 5 plays an important role in the immune processes of various infectious diseases, such as pathogen removal and inflammatory response regulation. These effects can limit the development of infectious diseases and maintain the stability of the body’s internal environment but can also cause pathological damage under certain conditions. Multiple studies on CCR5 and HIV were published in 1996, identifying CCR5 as an essential co-receptor for HIV entrance. After the envelope glycoprotein attaches to CCR5, the envelope is embedded in the host cell membrane (Alkhatib et al., 1996; Kwong et al., 1998). Neuronal damage caused by HIV infection leads about half of the infected people to acquire HIV-related neurocognitive disorders (Ru and Tang, 2017). Microglial activation and subsequent neuronal injury are prevented by the genetic deletion of CCR5, which in a transgenic model also rescues spatial learning and memory. Cognitive performance is also improved in chronic HIV patients after dual CCR2 and CCR5 antagonism (Maung et al., 2014; D’Antoni et al., 2018). CCR5 is crucial in West Nile virus (WNV) infection as an antiviral and survival factor, as evidenced by the enhanced leukocyte accumulation in the CNS and increased survival of up to 60% after splenocytes from WNV-infected WT mice were transferred into WNV-infected CCR5^–/–^ mice (Glass et al., 2005). Endothelial cells of the cerebral microvasculature produce CCL3, CCL4, and CCL5 after cerebral malaria infection, which could attract CCR5-positive leukocytes toward the brain, where they would eliminate parasites. Nevertheless, brain-recruited effector CD8^+^ T cells destroy ECs, causing the BBB to break (Belnoue et al., 2003).

### C–C Chemokine Receptor 5 and Cancers

C–C chemokine receptor 5 has anti-cancer and pro-cancer effects. Anti-cancer properties include recruitment of tumor-infiltrating lymphocytes and destruction of cancer cells. In contrast, chemokines exhibit pro-cancer properties by promoting angiogenesis and lymphangiogenesis, as well as enhancing cancer cell migration, invasion, and proliferation and recruiting cells that promote tumor development (Korbecki et al., 2020). CCR5 plays a role in tumor development or progression in multiple myeloma, classical Hodgkin lymphoma, prostate, breast, gastric, colon, and ovarian cancer, glioblastoma, and melanoma (Brunn et al., 2007; Yu-Ju Wu et al., 2020). Notably, CCR5 promotes tumor cell proliferation mechanisms, including the Jak-STAT or the MAPK/ERK signaling pathway leading to upregulation of cyclin expression and the PI-3K pathway resulting in the proliferation of progenitor and stem cells when the serine/threonine kinase protein kinase B (AKT) and PDK1 increase. Furthermore, via Akt phosphorylation, which stimulates the uptake of glucose, glutamine metabolism, fatty acid synthesis and the pentose phosphate pathway, CCR5 enables tumor cells to utilize glucose and catabolites more efficiently (Aldinucci and Colombatti, 2014; Jiao et al., 2019). CCR5 induces the expression and activity of DNA repair genes, resulting in aberrant cell survival and resistance to agents that cause DNA damage. The CCR5 antagonists Maraviroc and Vicriviroc dramatically enhance cell destruction mediated by DNA-damaging chemotherapeutic agents (Jiao et al., 2018).

### C–C Chemokine Receptor 5 and Pain

Spinal CCR5 is involved in the development and maintenance of pathological pain, including visceral hyperalgesia, cancer-induced bone pain, and neuropathic pain induced by spinal nerve injury. A novel medication for pathological pain that targets the CCL8/CCR5/ERK pathway in the spinal cord can be developed (Piotrowska et al., 2016; Hang et al., 2017; Lu et al., 2017). Activation of CCR5 in the brain significantly reduce the antinociceptive action of opioid receptor agonists, which is based on heterologous desensitization of μ-opioid receptors. These results shed light on the treatment of hyperalgesia related to inflammatory reactions and also suggest that the chemokine system, joins neurotransmitters and neuropeptides (Szabo et al., 2002).

### C–C Chemokine Receptor 5 and Other Central Nervous System Diseases

Alzheimer’s disease (AD) is a neurodegenerative disease that is characterized by a neuroinflammatory component. CCR5 and its ligands are overexpressed in both the periphery and brain of AD patients, which activates astrocytes and microglia, leading to amyloid deposits and memory dysfunction. However, some studies have also shown that CCR5 deletion can lead to worsening of AD, which may be due to a compensatory increase in CCR2 (Cartier et al., 2005; Goldeck et al., 2013). The autoimmune disease multiple sclerosis (MS) is a CNS disease with chronic inflammation caused by T cells. T cells from patients with MS had a considerably higher migratory rate than healthy cells that selectively migrated toward CCL3. In addition, CCR5 is involved in myelin degradation and, hence, plays a crucial role in the progression of MS (Zang et al., 2000; Janssen et al., 2016).

This review focuses on the research progress of CCR5 in ischemic stroke, but there are numerous functions of CCR5 in stroke and other diseases in CNS waiting for exploration. A recent study found CCR5 activation after intracerebral hemorrhage, partially through the CCR5/PKA/CREB/NLRP1 (nucleotide-binding domain leucine-rich repeat pyrin domain containing 1) signaling pathway, promoted neuronal pyroptosis, and neurological deficits (Yan et al., 2021).

## Conclusion

The broader biological role of CCR5 has been confirmed as scientific research progresses. CCR5 was once thought to be involved exclusively in immune responses, such as leukocyte migration and pathogen clearance, but new data have revealed that it also modulates cell signaling and neural plasticity, which play a role in the control of learning and memory. The therapeutic effect of promoting cerebral reperfusion after stroke is strictly limited by the therapeutic time window, and limited recovery after acute brain injury leads to the prevalence of disability after stroke; therefore, neurorehabilitative therapies have broad therapeutic prospects. Numerous molecular, cellular, and behavioral studies have been conducted on neural recovery after brain injury, and recent studies have demonstrated that the function of CCR5 signaling is essential in human stroke recovery. CCR5 activation reduces neuroplasticity and inhibits the recovery process after stroke through CREB/MAPK inactivation, impaired axonal regeneration, and decreased synaptic plasticity, whereas inhibition of CCR5 function promotes neurorehabilitation after stroke. CCR5, the first reported gene linked to improved neurological recovery after stroke in humans, reopens the recovery window after stroke (Zhou et al., 2016; Joy et al., 2019; Servick, 2019). There are common mechanisms between memory formation and brain repair, and the induction of neuronal plasticity provides a new therapeutic direction for promoting the recovery of motor and cognitive functions in patients with stroke.

## Author Contributions

Y-QF, Z-ZX, Y-TW, and YX contributed to the conceptual design, writing, and editing for this manuscript. Y-YH, LC, WLX, and G-YL conceived the tables. XL and JL provided critical input. QPW revised the manuscript and commented on previous versions of the manuscript. All authors contributed to the article and approved the submitted version.

## Conflict of Interest

The authors declare that the research was conducted in the absence of any commercial or financial relationships that could be construed as a potential conflict of interest.

## Publisher’s Note

All claims expressed in this article are solely those of the authors and do not necessarily represent those of their affiliated organizations, or those of the publisher, the editors and the reviewers. Any product that may be evaluated in this article, or claim that may be made by its manufacturer, is not guaranteed or endorsed by the publisher.

## Abbreviations

AC: adenylyl cyclase
AD: Alzheimer’s disease
AMPA: α-amino -3-hydroxy-5methyl-4-isoxazole propionic acid
BBB: blood-brain barrier
BDNF: brain-derived neurotrophic factor
cAMP: cyclic adenosine monophosphate
CCR5: C–C chemokine receptor 5
CNS: central nervous system
CREB: cAMP-response-element binding protein
DAG: diacylglycerol
DC: dendritic cells
DLK: dual-leucine zipper kinase
ERK1/2: extracellular signal-regulated kinase
GABA: gamma-aminobutyric acid
GPCR: G protein-coupled receptor
HIV: human immunodeficiency virus
ICAM-1: intercellular adhesion molecule-1
IP3: inositol 1,4,5-trisphosphate
JAK2: Janus kinases
JNK: C-JunN-terminal kinases
LPS: lipopolysaccharide
MAP: mitogen-activated protein
MAPK: mitogen-activated protein kinase
MMPs: matrix metalloproteinases
NMDAR-1: N-MethylD-Aspartate Receptor 1
NK: natural killer cell
pCREB: phosphorylated cAMP-response-element binding protein
PIP2: phosphatidylinositol 4,5-bisphosphate
PMCA: plasma membrane Ca^2+^-ATPase
PI-3K: phosphatidylinositol 3-kinases
PKB: protein kinase B
PLC: phospholipase C
PYK2: proline-rich tyrosine kinase 2
STAT: signal transducer and activator of transcription
TBI: traumatic brain injury
VCAM-1: vascular cell adhesion molecule 1.
